# The Antenatal and Postnatal Consequences of Antenatal Exposure to Prolonged Low Dose Indomethacin

**DOI:** 10.3390/jcm10091851

**Published:** 2021-04-24

**Authors:** Vera Donadono, Nicky Manning, Lawrence Impey

**Affiliations:** 1 Department of Fetal Medicine, John Radcliffe Hospital, Oxford University Hospitals NHS Foundation Trust, Oxford OX3 9DU, UK; lawrence.impey@ouh.nhs.uk; 2 Department of Fetal Cardiology, John Radcliffe Hospital, Oxford University Hospitals NHS Foundation Trust, Oxford OX3 9DU, UK; nicky.manning@gosh.nhs.uk

**Keywords:** indomethacin, ductus arteriosus, fetal echocardiography, pregnancy, preterm delivery

## Abstract

Despite its many clinical applications, indomethacin is seldom used in pregnancy, principally because of concerns regarding the potential for constriction of the arterial duct. The aim of this study was to document adverse antenatal effects and postnatal outcomes after in utero exposure to low-dose indomethacin. We studied a retrospective cohort of pregnancies between 2005 and 2016 at the John Radcliffe Hospital, Oxford, UK, in which mothers at extremely high risk of preterm birth were treated as prophylaxis with indomethacin 25 mg, 12 hourly, before 29 weeks. Antenatal effects on the arterial duct and postnatal outcomes were analysed. Overall, 198 fetuses had in utero follow-up, and 13 (6.6%) had ductal constriction, all within 9 days of starting treatment. No ductal constriction was seen in pregnancies when therapy was started before 20 weeks, and all effects were reversed after cessation of therapy. An analysis of postnatal complications was possible in 181 neonates. There were eight (4.4%) neonatal deaths, all but one associated with extreme preterm birth. Seven (5%) patent ductus arteriosus cases occurred in the 140 neonates delivered after 28 weeks who were alive at discharge. Postnatal complications were not more common in neonates in whom antenatal ductal constriction had been demonstrated. In conclusion, fetuses exposed to prolonged low dose indomethacin have a low incidence of in utero complications; these complications can be diagnosed with ultrasound and are reversible. Adverse postnatal events are related to gestation at birth and do not appear more common.

## 1. Introduction

Prostaglandin synthetase inhibitors, including indomethacin, are recognised to be an effective therapy for delaying preterm labour by 48 h–7 days, according to a recent meta-analysis [1]. Indomethacin has also been used in women with a short cervix [2] and at the time of and following cervical cerclage [3,4]. It is also used at the time of therapeutic in utero interventions [5]. Despite its potential uses, indomethacin is seldom prescribed in pregnancy because of antenatal concerns of the ductus arteriosus (DA) constriction, although the risk of this remains unclear. The aim of this study was to document the ductal effects of a large cohort of babies who had in utero exposure to indomethacin.

## 2. Materials and Methods

This is a retrospective cohort study of pregnancies between January 2005 and June 2016 at the John Radcliffe Hospital, Oxford, UK, in which the mother was treated with indomethacin with the aim of reducing the risk of extreme preterm labour. Echocardiographic details were collected prospectively; other details were collected from antenatal and neonatal records. Pregnancies were excluded from analysis for the following criteria: (1) if a major abnormality was detected (either ante or postnatally), (2) if treatment was given because of polyhydramnios in view of its association with various genetic disorders, infections, twin-to-twin transfusion syndrome and other rarer causes, (3) if they delivered before any echocardiography could be performed, and (4) if they were lost to follow up. Demographic, obstetric and neonatal details were recorded.

Women received indomethacin if considered at very high risk of extreme preterm delivery, usually in addition to cervical cerclage. In these women, indomethacin was usually stopped by 30 weeks. Indomethacin was used at a dose of 25 mg twice a day, for at least 72 h.

Fetal echocardiography was performed using a Siemens Acuson from 2005 to 2012 and a GE Voluson E8 after that. The examination was initially performed at 3–7 days, repeated within 14 days following indomethacin exposure, and then twice weekly, if abnormal, during the course of treatment. If the result was normal, no structured cardiologic follow-up was made. Growth scans were performed on a case-by-case basis. Fetal echocardiography included assessment of cardiac anatomy, evidence of right heart dilatation, presence of tricuspid regurgitation, and measurement of the pulsatility index (PI) of the DA. This was obtained using either a 3-vessel view or sagittal view, depending on the fetus’s position. The DA was demonstrated on a 2D image and with colour Doppler, and the PI was measured using pulsed-wave Doppler. A PI of less than 2.0 was considered suspicious of evidence of ductal constriction [6]. If the PI was 1.7–2.0, follow-up continued. If the PI was 1.7 or below, indomethacin therapy was stopped.

Antenatal analysis was of in utero effects of indomethacin administration on the DA. Postnatal analysis was of in utero evidence of ductal constriction correlated to postnatal adverse outcomes.

Results are expressed as numerical values with percentages for categorical variables and continuous variables as mean ± standard deviation, or median and range when not normally distributed. A chi-square test and Student’s *t*-test, or fisher’s exact test and Mann–Whitney U test were carried out as appropriate; odds ratio (OR) and 95% confidence interval (CI) were presented to explore antenatal and postnatal adverse outcomes. A *p*-value < 0.05 was considered statistically significant. Data were analysed using the statistical package SPSS Statistics version 25.0 (IBM Corp., Armonk, NY, USA). The study was performed according to “The Strengthening the Reporting of Observational Studies in Epidemiology” [7].

## 3. Results

**Figure 1:**
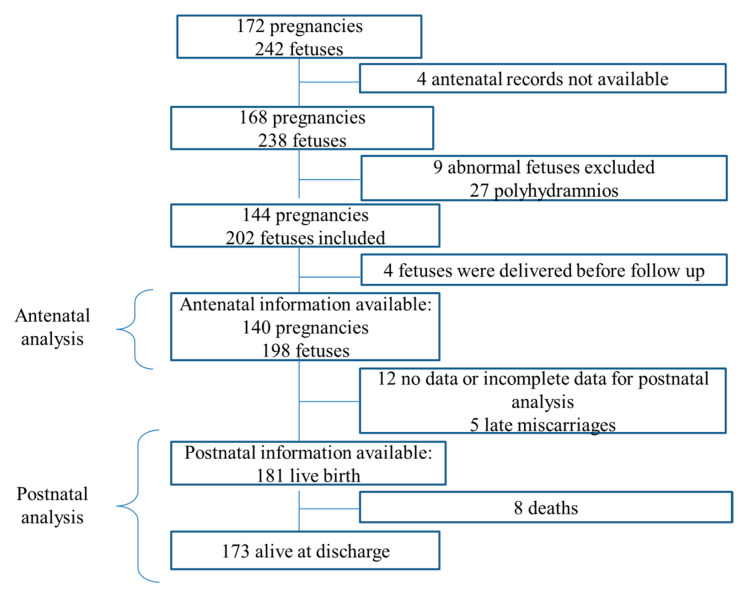
Flow-chart of inclusions and exclusions. Antenatal analysis refers to the cohort of patients included for the study of the antenatal complications. Postnatal analysis refers to the cohort of patients included for the study of the postnatal outcome.

Between January 2005 and June 2016, in 172 women, including 242 fetuses, indomethacin was started before 29 weeks gestation. In four women, antenatal records were not available, nine neonates had major malformations, and four pregnancies ended before in utero surveillance was instigated. In a further 27, indomethacin was used for the treatment of polyhydramnios. These fetuses were all excluded. A total of 140 pregnancies, including 198 fetuses (94 singletons, 37 sets of twins, 7 sets of triplets, 1 set of quintuplets, and 1 set of sextuplets) had in utero follow-up (Figure 1) and were included in the antenatal analysis. For postnatal analysis, there were 181 neonates (Figure 1). Of the other 17, there were 5 late miscarriages, and in 12 cases, delivery occurred elsewhere in the third trimester with live-born, but detailed neonatal data were unavailable.

Maternal and pregnancy characteristics of included women receiving indomethacin and duration of therapy are shown in Table 1. Indomethacin was given to reduce the risk of preterm birth: in most, this accompanied cervical cerclage.

### 3.1. Antenatal Analysis

In 13 (6.6%) fetuses, there were detectable ductal effects, all detected within 9 days (Table 2): no ductal constriction was subsequently seen in fetuses who were unaffected at this stage. In two (15.4%), the PI of DA was <1.7, and indomethacin was discontinued. In the other 11 (84.6%), the PI was between 1.7–2.0, indomethacin was continued, and the DA was observed. In these, after a period of 2–10 days, the PI normalised in 9/11 fetuses (81.8%) despite continuation and remained abnormal in 2/11 fetuses (18.2%), and so indomethacin was stopped. Of the four pregnancies where indomethacin was stopped, it was restarted in one: follow-up showed no following ductal constriction. In no case was cardiac failure observed. Moderate tricuspid regurgitation was present in only one fetus with transient constriction and was resolved following normalisation of the ductal PI.

7.4% (7/94) of fetuses of singleton pregnancies and 5.8% (6/104) of fetuses of multiple pregnancies had ductal constriction, and this difference was not significant (*p* = 0.634).

The gestation at starting indomethacin was significantly higher in fetuses where DA constriction had been seen (median 23.43 weeks; range 20.29–28 weeks) than where it had not (median 22 weeks; range 12–28.43 weeks) (*p* = 0.03). Indeed, none of the 71 (35.85%) fetuses exposed before 20.14 weeks developed ductal constriction.

### 3.2. Postnatal Analysis

Postnatal outcomes are shown in Table 3. Neonatal deaths happened at a time after birth that varied from less than a day to 16 days after delivery. Seven deaths occurred in neonates delivered before 28 weeks and one death in a baby born at 28.57 weeks due to sepsis and pneumothorax. All these children have been excluded from the postnatal analysis of patent ductus arteriosus (PDA) because not enough time had passed after birth to allow for duct assessment. No deaths were due to PDA. In 27 out of 33 (81.8%) babies delivered before 28 weeks, there was evidence of PDA; seven (25.9%) of these underwent surgery. PDA was also diagnosed in 7 (5%) neonates born beyond 28 weeks, none of whom required surgery. Postnatal ductal complications were not common where there had been antenatal ductal effects. Moreover, no significant difference was found between the two groups in the incidence of intraventricular haemorrhage grade > 2, bronchopulmonary dysplasia, renal failure, and noo case of cardiac failure was reported at discharge.

## 4. Discussion

This paper describes the incidence of ductal complications in 198 anatomically normal fetuses exposed to low dose indomethacin to prevent severe preterm birth.

The majority of the existing literature regarding antenatal effects of indomethacin is summarised by Abou-Ghannam et al. [8], but ductal constriction varies from 6.5 to 80% [9,10]. However, the series was small, doses were variable, and the gestation was often late. Ductal constriction from more prolonged therapy, albeit mostly commencing later than in our series and in varying doses, is addressed by Savage et al. in a series of 124 exposed pregnancies [9]. In contrast to this series, we found that the gestational age when starting therapy is important, with no effects seen in fetuses exposed before 20 weeks. From our results, it appears that the duration of indomethacin treatment did not influence the DA when administered for more than 9 days, as all cases of ductal constriction happened in the first 9 days. We cannot exclude the possibility that ductal constriction occurred temporarily later in pregnancy, but no cases of heart failure were observed, either in utero or postnatally, in the entire cohort. We also demonstrated transient ductal constriction that can resolve despite the continuation of therapy. No difference in terms of ductal effect was found when comparing fetuses of singleton and multiple pregnancies.

It has also been suggested that antenatal indomethacin increases failure rates of postnatal medical therapy [11,12]. Our data suggest that antenatal ductal constriction does not influence the incidence or severity of postnatal ductal complications and neonatal outcome.

This is the largest series of fetuses in utero exposed to indomethacin; all women received the same dosage, and detailed outcome data were available for the majority. This study is limited by its retrospective nature, although antenatal ductal data were recorded prospectively. All pregnancies were at increased risk of preterm birth, as reflected in the perinatal outcomes, yet this is the group where antenatal indomethacin is most likely to be useful. The dose of indomethacin is relatively small, was used for variable and sometimes prolonged time frames, and it is acknowledged that transient effects could have occurred prior to echocardiography. While controls could have been used to analyse postnatal complications, these would have needed to be matched for gestational age, at least in the extreme preterm group: such babies should not be considered good controls, as the final result would not be reliable [13]. Analysis of postnatal ductal complications is also limited by consensus regarding treatment [14] and because the thresholds for intervention varied during our timeframe.

In conclusion, we describe the incidence, timing, and nature of antenatal DA constriction and its postnatal sequelae. Fetuses exposed to prolonged low dose indomethacin from before the third trimester have a low incidence of in utero complications; these complications happened in the first 9 days of treatment, can be diagnosed with ultrasound, and are reversible. Adverse postnatal events are related to gestation at birth and do not appear more common. These data provide some reassurance regarding indomethacin usage in daily practice and clinical research, and this should enable further investigation of the potential of non-steroidal drugs as prophylaxis against preterm birth.

## Figures and Tables

**Table 1:** Pregnancy characteristics.

Maternal Characteristics	
Total	140
Age, years	31.95 (16.75–46.71)
Booking BMI, kg/m^2^	23.48 (17.15–46.25)
Smoker	10/109 (9.17)
Ethnicity white	85/114 (74.56)
Nulliparous	84/135 (62.22)
Multiple pregnancy	46 (32.86)
**Indomethacin Indication**	
Cerclage	124 (88.57)
*Rescue*	54 (43.55)
*USS Indicated*	41 (33.07)
*Elective*	29 (23.39)
Short Cervix but No Cerclage	16 (11.43)
**Indomethacin Administration Characteristics**	
GA at starting indomethacin, w + d	22 + 0 (12 + 0–28 + 3)
GA at suspension indomethacin, w + d	28 + 0 (16 + 5–33 + 3)
Indomethacin duration, days	39 (7–130)
Indomethacin interval last dose to delivery, w + d	6 + 2 (0–20 + 4)

Notes: GA = gestational age; w + d = weeks + days; USS = ultrasound. Data are expressed as frequencies (percentage) or median (range). Italic identifies subgroups.

**Table 2:** Antenatal ductal effects of indomethacin.

**Total**				198
**DA Constriction**				13/198 (6.6)
	Mild (PI 1.7–2.0)			11/13 (84.6)
		Normalised		9/11 (81.8)
		Persistent		2/11 (18.2)
	Severe (PI < 1.7)			2/13 (15.4)
		Stopped		4/13 (30.8)
		Restarted		1/4 (25)
			Normalised	1/1 (100)
			Persistent	0/1 (0)
**Tricuspid Rigurgitation**				1/198 (0.5)

Notes: DA = ductus arteriosus; PI = pulsatility index. Data are expressed as frequencies (percentage).

**Table 3:** Adverse pregnancy outcomes, and according to ductal constriction.

	Total	No Antenatal DA Constriction	Antenatal DA Constriction	*p* Value	OR (95% CI)
**Total Number of Fetuses**	**198**	**185 (93.4)**	**13 (6.6)**		
In utero loss < 23 + 0	5 (2.5)	5 (2.7)	0	1 *	
Lost to neonatal follow-up	12 (6.1)	12 (6.5)	0	1 *	
**Total Number of Neonates**	**181**	**168 (92.8)**	**13 (7.2)**		
GA at delivery, weeks + days	34 + 2 (23 + 1–41 + 1)	34 + 2 (23 + 1–41 + 1)	33 + 6 (24 + 2–40 + 2)	0.820 **	
N deliveries < 37 + 0	134 (74)	126 (75)	8 (61.5)	0.327 *	1.86 (0.58–6.05)
N deliveries < 32 + 0	77 (42.5)	72 (42.9)	5 (38.5)	0.757	1.2 (0.38–3.82)
N deliveries < 28 + 0	40 (22.1)	35 (20.8)	5 (38.5)	0.165	0.42 (0.13–1.37)
Birthweight, grams	1905 (510–4217)	1900 (560–4217)	2396 (510–3858)	0.714 **	
Admission to NICU	114 (63)	106 (63.1)	8 (61.5)	1 *	0.94 (0.29–2.99)
Neonatal death	8 (4.4)	7 (4.2)	1 (7.7)	0.456*	1.92 (0.22–16.89)
**Total number of alive neonates at discharge**	**173**	**161 (93.1)**	**12 (6.9)**		
IVH, grade > 2	4 (2.3)	4 (2.5)	0	1 *	
BPD	27 (15.6)	24 (14.9)	3 (25)	0.403 *	1.90 (0.48–7.54)
Renal failure	1 (0.6)	1 (0.6)	0	1 *	
Any PDA	34 (19.7)	30 (18.6)	4 (33.3)	0.255 *	2.18 (0.62–7.73)
Resolved spontaneously	5 (14.7)	4 (13.3)	1 (25)	0.488 *	2.17 (0.18–26.29)
Medical treatment	22 (64.7)	19 (63.3)	3 (75)	1 *	1.74 (0.16–18.8)
Surgical ligation	7 (20.6)	7 (23.3)	0	0.559 *	

Notes: DA = ductus arteriosus; OR = odds ratio; CI = confidence intervals; GA = gestational age; NICU = neonatal intensive care unit; IVH = intraventricular haemorrhage; BPD = bronchopulmonary dysplasia; PDA = patent ductus arteriosus. Bold identifies a group. * Fisher’s exact test; ** Mann–Whitney U test.

## Data Availability

The data presented in this study are available on request from the corresponding author.
